# Syphilis causing elevation of anti-CCP: a case report of medical mimicry

**DOI:** 10.3389/fmed.2026.1952312

**Published:** 2026-09-03

**Authors:** Tara Subrahmanyan, Ashley Martinez, Justin Baik, Matthew Kelly

**Affiliations:** Department of Medicine, Western Michigan Homer Stryker MD School of Medicine, Kalamazoo, MI, United States

**Keywords:** anti-CCP, penicillin, rheumatoid arthritis, syphilis, Treponema pallidum

## Abstract

**Introduction:**

Infection with Treponema pallidum, or syphilis, is often called the “Great Imitator” because of its propensity for atypical presentations and non-specific symptoms. In addition, syphilis has been shown to create false-positive results for common tests, including Lyme disease, HIV, and multiple autoimmune conditions. One that has not been documented is anti-cyclic citrullinated peptide (anti-CCP). Anti-CCP is a highly specific test for Rheumatoid Arthritis. However, in this case, syphilis led to false-elevations in anti-CCP.

**Case:**

A 29-year-old G5P3 female presented to the emergency department with a 3-month history of progressive rash, night sweats, chills, lymphadenopathy, dysphagia, headache, and arthralgia. Work-up included a positive CMV test and elevated C-reactive protein and Erythrocyte Sedimentation Rate. Remaining tests were normal, and she was treated symptomatically for CMV. Eventually, her dysphagia progressed to an inability to tolerate liquids, prompting hospitalization. Examination showed a diffuse rash, including the palms and soles, and painful lymphadenopathy. Laboratory testing revealed positive rapid plasma reagin, confirmed by a treponemal antibody study. She was initially treated with doxycycline due to penicillin shortages, with plans to receive penicillin at the health department. However, she returned four days later with neurological symptoms and results positive for neurosyphilis and was successfully treated with a 14-day course of intravenous penicillin. During her first admission, results were positive for anti-CCP and antinuclear antibodies (ANA), suggesting a rheumatologic etiology. Six months later, follow-up studies showed resolution, suggesting syphilis caused the elevations.

**Conclusion:**

Secondary syphilis is typically associated with the elevation of certain autoimmune markers; however, anti-CCP is not commonly among them. This particular case is notable due to the elevated anti-CCP level observed in the absence of any rheumatologic disease. In alignment with the majority of secondary syphilis cases, the patient presented with significant joint pain and swelling as the primary symptoms. This clinical manifestation, coupled with positive ANA and anti-CCP results, could easily result in a misdiagnosis of rheumatoid arthritis. If left untreated, syphilis may advance to neurosyphilis, leading to severe complications. This case is significant as it underscores a novel instance of medical mimicry in secondary syphilis, which clinicians must be able to identify.

## 1 Introduction

Syphilis, an infection caused by Treponema pallidum, is a complex disease that poses a diagnostic challenge for healthcare providers. It is often referred to as the “Great imitator” because it typically presents with vague and confounding clinical features that are present in different pathologies (1). The United States has experienced a resurgence of syphilis over the last 25 years, which has now reached epidemic proportions. In 2000-2001, syphilis rates were as low as 2.1 per 100,000 people. However, these numbers increased to 62.5 per 100,000 in 2023 (2). This represents a 30-fold increase over 22 years. A consequence of these statistics is the congenital syphilis crisis, which has seen a 10-fold increase in rates. As there are continued shortages of benzathine penicillin, the primary treatment, syphilis represents an extreme threat to public health in the US (1). This case report documents a particularly difficult diagnosis of syphilis. It highlights a positive test that is not typically associated with syphilis: anti-cyclic citrullinated peptide. Antibodies against cyclic citrullinated peptides (anti-CCP) have been shown to be highly specific to rheumatoid arthritis (RA) and other autoimmune diseases. However, there is only one case linking it to syphilis. This case report is important because it suggests that anti-CCP may be associated with a diagnosis of syphilis in future patients.

Treponema pallidum is a spirochete that is predominantly transmitted via sexual contact and vertically during pregnancy. Screening is recommended at least once in a lifetime for all individuals with risk factors and annually for men who have sex with men, individuals with human immunodeficiency virus (HIV), and transgender individuals. It is also recommended every 3−6 months for individuals receiving HIV pre-exposure prophylaxis (1, 2). However, as Treponema pallidum does not gram stain, testing can be challenging. The current gold standard for testing uses direct detection, typically via dark-field microscopy. However, serological studies are often used to diagnose this condition. Typical testing starts with an initial nontreponemal test (NTT), such as the RPR or venereal disease research laboratory (VDRL) test. After one of these tests is positive, it is confirmed with a treponemal test, such as the fluorescent treponemal antibody absorption (FTA-ABS) or Treponema pallidum particle agglutination (TPPA) test. Treponemal tests are based on the qualitative detection of treponemal antibodies, which leads to higher sensitivity and specificity than nontreponemal tests (1, 2).

Syphilis has four stages: Primary Syphilis occurs at the onset of infection, presenting with a genital ulcer or rash that is often painless. This can create challenges because patients, such as in this case, may believe it to be harmless or may not notice it at all. However, once a patient is inoculated with the bacteria, it can enter a latent stage and progress to secondary syphilis. Secondary Syphilis presents with headache, tearing, nasal secretions, pharyngitis, generalized myalgias, arthralgias, and a maculopapular cutaneous rash that typically involves the palms and soles (1, 2). Primary, secondary, and early latent stages are treated with a single 2.4 million unit intramuscular (IM) dose of benzathine penicillin G (3). Without proper treatment, syphilis can re-enter the latent stage and eventually progress to tertiary syphilis, which is treated with three doses of IM penicillin, resulting in a total of 7.2 million units (1–3). Tertiary syphilis typically occurs after a 10–30 year period of latency and can present differently depending on the site of infection. Soft tissue infection can result in gummatous syphilis, and cardiovascular syphilis can occur when seeded in the body's vasculature. One major concern in tertiary syphilis is damage to the nervous system, which results in neurosyphilis. This can lead to dementia, loss of motor control, numbness, blindness, hearing loss, and tabes dorsalis (1, 2). Neurosyphilis also requires an additional 14 day course of intravenous (IV) penicillin (18–24 million units a day) prior to three weeks of IM treatment (3). In cases of penicillin shortage or allergy, the best-supported alternative is doxycycline, which has a high failure rate (3). Overall, syphilis represents an extreme threat to public health in the US, and it is important that physicians are prepared to manage it.

## 2 Case

A 29-year-old woman (G5P3) with no known medical history presented to the emergency department with cytomegalovirus (CMV) infection and progressive viral symptoms. The patient reported a 3-month history of progressive rash, night sweats, chills, lymphadenopathy, dysphagia, headache, and arthralgia. The patient also reported new-onset diarrhea. She had a history of a positive CMV test and was being treated symptomatically. However her pharyngitis progressed to her being unable to tolerate solids or liquids, which led to her admission to the medicine team. The patient was started on fluids for dehydration. She initially presented with a blood pressure of 106/71 mmHg, pulse of 108 bpm, temperature of 37.4 °C, and oxygen saturation of 98%. Physical examination revealed a rash throughout her body, including the palms and soles. She had areas of solitary nodules that were not grouped or dermatomally located. The lesions were located on her face, neck, and back, with two on her right wrist. She also reported diffuse painful lymphadenopathy and arthralgia. Physical examination also revealed generalized joint swelling and bilateral knee tenderness. Multiple laboratory tests were performed upon admission. These tests revealed a positive RPR (1:128) test result. This was followed by a positive treponemal antibody test. The patient was diagnosed with early secondary syphilis. Simultaneously, the patients' laboratory studies also resulted in positive anti-CCP (45) and ANA (1:320) results. Her other rheumatologic studies were negative, such as rheumatoid factor. This was highly suggestive of an early rheumatologic disease. However, since she had a confirmed case of syphilis, which can quickly lead to much more severe complications, it was determined that the patient should be treated for syphilis first. Once she was treated, a rheumatologic work up was planned outpatient as the team was unable to discriminate which symptoms may be due to syphilis versus a rheumatologic etiology. Due to nationwide shortages, the patient was prescribed doxycycline and instructed to follow up with her local health department to receive a subcutaneous dose of penicillin.

Four days after discharge, the patient presented to the health department for a subcutaneous penicillin injection. While there, she reported vision changes, significant weakness, and enhanced soreness of her throat and jaw. The health department referred her to the emergency department due to concerns of neurosyphilis, and the patient was readmitted to the same service. On initial workup, she was found to have a positive Neurosyphilis IgG antibody index and vision changes. Due to the positive neurosyphilis antibody test result and neurological symptoms, a neurosyphilis protocol was initiated, and the patient was started on intravenous penicillin for 14 days. She received 18–24 million units daily via IV and peripherally inserted central catheter, and therapeutic success was determined by a reduction in her repeat RPR (1:32). Other studies performed during her hospitalization included a lumbar puncture and CSF analysis, which showed seven nucleated cells, a normal gram stain and culture, and a negative venereal disease research laboratory (VDRL) test. Six months after hospitalization, the patient underwent repeat rheumatological studies as instructed. These tests included anti-CCP, C-reactive protein (CRP), and erythrocyte sedimentation rate (ESR) tests. All of these laboratory values returned to normal, and the patient reported no associated symptoms.

**Figure 1:**
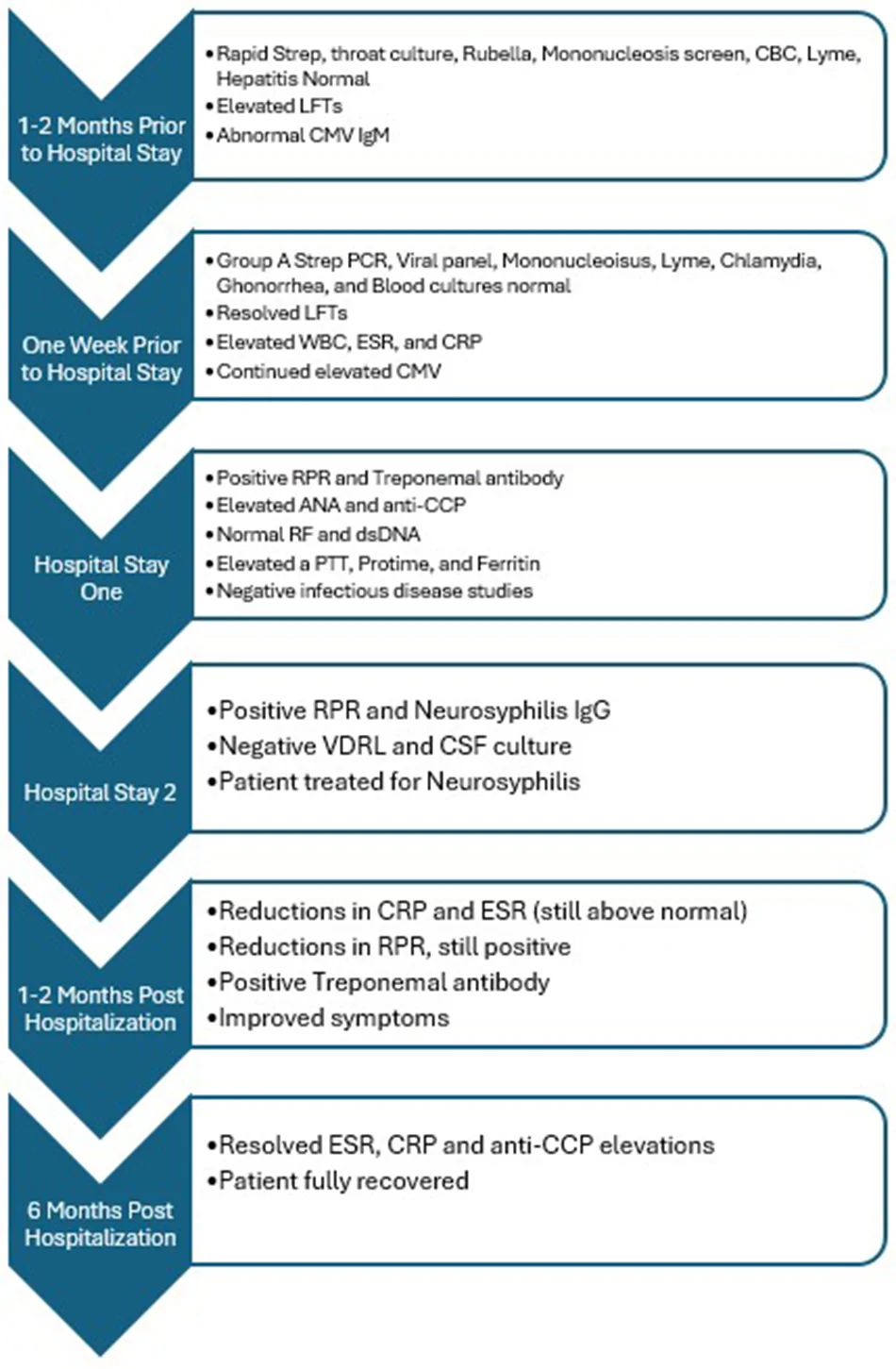
Timeline of patients key laboratory studies, results, and important clinical events.

Two months prior to hospitalization, the patient presented to her primary care provider (PCP) with similar symptoms. Her initial workup included a rapid streptococcus A test with throat culture, a rubella IgM test, Lyme antibody screen, and mononucleosis screen. The patient also underwent complete blood count. At that time, all tests were within normal limits. She was treated symptomatically and advised to return if her symptoms persisted. A month later, she presented to her PCP again. At that time, she had a normal thyroid function and hepatitis panel. However, her CMP showed elevated liver markers (LFTs), aspartate aminotransferase (AST) and alanine aminotransferase (ALT). The patient also tested positive for CMV. Around the same time, her husband tested positive for mononucleosis with Epstein–Barr virus (EBV). Therefore, her provider suspected mononucleosis due to CMV infection. The patient was advised to undergo retesting after 14 days of symptomatic treatment. However, her symptoms continued to progress, and 8 days later, she presented to the emergency department. At this time, the patient was tested for Group A streptococcus, Lyme disease, Chlamydia, and gonorrhea. She also underwent a full viral panel and blood culture, all of which were normal. Her CMP also showed resolution of the elevated liver enzymes. At this time, she was found to have elevated CRP (184) and ESR (85) levels. Thus, the patient was instructed to follow up with rheumatology and infectious disease outpatient, and was discharged. However, her symptoms progressed, leading to hospitalization and subsequent diagnosis of syphilis. Figure 1. Represents patients clinical timeline.

Table 1. Important laboratory results during the patients clinical course

**Table 1:** Important laboratory results during the patients clinical course.

Lab	1-2 Months prior to hospital	One week prior to hospital	Hospital stay one	Hospital stay two	1-2 Months post hospital	6 Months post hospital
CMV (AI)	0.9	1.2				
CRP (mg/L)		184.0			17.2	<3.0
ESR (mm/h)		85			50	1
Anti CCP (U/mL)			45			<8.0
RPR			1:128	1:128	1:32	
Treponemal Antibody			Positive		Positive	
ANA			1:320			
RF/dsDNA [(Ui)/mL]			12/1.0			
aPTT (s)			44			

## 3 Discussion

One of the most distinctive features of this case was that the patient had elevated antibodies to cyclic citrullinated peptides. Citrulline is a peptide that is made when the amino acid arginine undergoes citrullination. This typically occurs when the body undergoes inflammatory processes or experiences a normal cell turnover. However, in some individuals, the body can misidentify the protein, leading to an immune response that generates antibodies against it. These antibodies are strongly associated with the diagnosis of rheumatoid arthritis (RA). The test has a specificity of 96% and a positive likelihood ratio of approximately 14 (4, 5). Thus, it can be very helpful in diagnosing RA. However, it is present in only approximately one-quarter to one-half of patients at the time of diagnosis. Therefore, it is much less sensitive, with a sensitivity of 53%–71% (4, 5). Although it cannot be used to rule out RA, its significant association with the disease makes positive results highly indicative of RA. This case is unique because anti-CCP levels were elevated in the absence of RA. In addition, once a patient has an elevated anti-CCP level, they tend to have it for the rest of their life. Levels may diminish slightly during treatment as inflammation is reduced, but they remain above normal limits. This case is uncommon because the patient's levels returned to normal 6 months after treatment (4, 5).

Although syphilis is not associated with elevated anti-CCP levels, there are multiple other non-RA causes of elevated anti-CCP levels. First, multiple rheumatologic diseases are associated with elevated anti-CCP levels. In a cohort of 1,162 patients, the prevalence of positive anti-CCP in patients with primary Sjögren's syndrome was 33.3% (6). Another disorder, systemic lupus erythematosus (SLE), was 16.6%. Other associated conditions examined in this study included juvenile idiopathic arthritis (15.9%), psoriatic arthritis (10.6%), mixed connective tissue disease (10.7%), and systemic scleroderma (8.3%). However, the presence of anti-CCP antibodies does not appear to indicate the future development of RA. In another study of 33 patients with non-RA-positive anti-CCP connective tissue diseases, two patients developed RA (6.1%) over a mean follow-up period of 8.9 years (7). The other causes of elevated anti-CCP levels are often infectious. These include HIV, tuberculosis, hepatitis B/C, and streptococcal infection (8). These are often associated with some level of arthritis. All of these are non-RA conditions that have been documented to elevate anti-CCP.

In addition to anti-CCP, multiple laboratory studies have shown abnormal labratory levels in syphilis infection. One of these is alkaline phosphatase or gamma-glutamyl transferase (GGT) activity. This has been shown to be markedly elevated in a cholestatic pattern during secondary syphilis (9). Consequently, elevated bilirubin levels can occur due to liver involvement in patients with syphilis. In addition to hepatic involvement, syphilis increases the levels of various inflammatory markers such as ESR and CRP, just as in this case. Other positive studies associated with syphilis include anticardiolipin antibodies, prolonged aPTT (with positive autoantibodies), rheumatoid factor, and ANA (10, 11). Prolonged aPTT and ANA were also present in our case. Additionally, patients may also have false-positive ELISA results for Lyme disease (12). These elevations have been attributed to molecular mimicry through structural homology in fibronectin, as well as polyclonal B-cell activation through bystander activation (13–15).. Altogether, syphilis can cause many laboratory abnormalities, but with more emerging, it is important for clinicians to be cognizant of these changes.

A crucial modifier in the clinical course of this patient was the limitations in treatment due to the current shortages of penicillin. Penicillin shortages have affected both high- and middle-to-low-income countries. A WHO-commissioned survey of 95 countries from to 2014–2016 found that 41% of countries experienced a shortage (16). In this case, the hospital was affected by penicillin shortages, and the patient was unable to receive penicillin while hospitalized. Instead, she was prescribed doxycycline and instructed to receive penicillin at her local health department. As discussed previously, doxycycline is the most supported alternative; however, it still has an extremely high failure rate (17).Consequently, by the time the patient reached the health department, she had developed signs of neurosyphilis. As seen in this case, the delay of definitive therapy can result in a rapid escalation of the disease, which can have long term consequences. This case represents the negative impact of the continued penicillin shortage experienced by patients and further need for the shortage to be addressed.

This case is important because it demonstrates how abnormal rheumatologic laboratory results can lead to the misdiagnosis of a condition that cannot be missed. To date, there has been only one other recorded case of elevated anti-CCP levels in a patient with syphilis. In this conference abstract, the patient, a 31-year-old male, primarily reported joint pain and stiffness (18). He had slightly elevated anti-CCP levels of 21 (normal <20) along with increased ESR (64) and CRP (24). In contrast, our patient showed an anti-CCP of 45, ESR of 85, and CRP of 184. Similar to our case, the patient in the abstract developed eye symptoms and was eventually treated for neurosyphilis (18).. Although the anti-CCP levels were just above the normal range, this abstract highlights a case that suggests that this elevation might not be an isolated incident but a growing trend that clinicians should be aware of. This also calls into question the mechanism which could be causing this, molecular mimicry, polyclonal B-cell activation, or another cause. Physicians should consider syphilis in the differential diagnosis when treating sexually active patients with elevated anti-CCP levels. As the syphilis crisis continues, it is crucial for physicians to recognize a wider range of presentations to ensure timely diagnosis and reduce the infection rates.

## Data Availability

The original contributions presented in the study are included in the article/Supplementary Material, further inquiries can be directed to the corresponding author.
